# Heterogeneity of cellular inflammatory responses in ageing white matter and relationship to Alzheimer’s and small vessel disease pathologies

**DOI:** 10.1111/bpa.12928

**Published:** 2021-02-15

**Authors:** Rachel Waller, Ruth Narramore, Julie E. Simpson, Paul R. Heath, Nikita Verma, Megan Tinsley, Jordan R. Barnes, Hanna T. Haris, Frances E. Henderson, Fiona E. Matthews, Connor D. Richardson, Carol Brayne, Paul G. Ince, Raj N. Kalaria, Stephen B. Wharton, CFAS

**Affiliations:** ^1^ Sheffield Institute for Translational Neuroscience University of Sheffield Sheffield UK; ^2^ Translational and Clinical Research Institute University of Newcastle Newcastle upon Tyne UK; ^3^ Institute of Public Health University of Cambridge Cambridge UK

**Keywords:** dementia, epidemiological neuropathology, neuroinflammation, small vessel disease, white matter lesions

## Abstract

White matter lesions (WML) are common in the ageing brain, often arising in a field effect of diffuse white matter abnormality. Although WML are associated with cerebral small vessel disease (SVD) and Alzheimer’s disease (AD), their cause and pathogenesis remain unclear. The current study tested the hypothesis that different patterns of neuroinflammation are associated with SVD compared to AD neuropathology by assessing the immunoreactive profile of the microglial (CD68, IBA1 and MHC‐II) and astrocyte (GFAP) markers in ageing parietal white matter (PARWM) obtained from the Cognitive Function and Ageing Study (CFAS), an ageing population‐representative neuropathology cohort. Glial responses varied extensively across the PARWM with microglial markers significantly higher in the subventricular region compared to either the middle‐zone (CD68 *p* = 0.028, IBA1 *p* < 0.001, MHC‐II *p* < 0.001) or subcortical region (CD68 *p* = 0.002, IBA1 *p* < 0.001, MHC‐II *p* < 0.001). Clasmatodendritic (CD) GFAP^+^ astrocytes significantly increased from the subcortical to the subventricular region (*p* < 0.001), whilst GFAP^+^ stellate astrocytes significantly decreased (*p* < 0.001). Cellular reactions could be grouped into two distinct patterns: an immune response associated with MHC‐II/IBA1 expression and CD astrocytes; and a more innate response characterised by CD68 expression associated with WML. White matter neuroinflammation showed weak relationships to the measures of SVD, but not to the measures of AD neuropathology. In conclusion, glial responses vary extensively across the PARWM with diverse patterns of white matter neuroinflammation. Although these findings support a role for vascular factors in the pathogenesis of age‐related white matter neuroinflammation, additional factors other than SVD and AD pathology may drive this. Understanding the heterogeneity in white matter neuroinflammation will be important for the therapeutic targeting of age‐associated white matter damage.

## Graphical abstract

Although white matter lesions (WML) are associated with cerebral small vessel disease (SVD) and Alzheimer’s disease (AD), their cause and pathogenesis remain unclear. Glial responses vary extensively across the parietal white matter (PARWM) with diverse patterns of white matter neuroinflammation. Understanding the heterogeneity in white matter neuroinflammation will be important for the therapeutic targeting of age‐associated white matter damage.

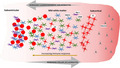

## 1 INTRODUCTION

White matter lesions (WML) are a common feature of ageing, found in over 90% of people over 65 years old (1). WML are identified in life by high signal intensities on T2‐weighted and diffusion tensor magnetic resonance imaging (MRI) (2). They are also considered as the radiological signature of cerebral small vessel disease (SVD) (3). However, WML are independently associated with cognitive impairment and risk of dementia (4, 5, 6), are also associated with depression (7) and impaired mobility (8) suggesting their significance in old‐age morbidity. Yet, their pathogenesis is complex and remains poorly defined.

Based on their anatomical location, WML can be defined as either periventricular lesions (PVL) located in white matter immediately next to the ventricles, or deep subcortical lesions (DSCL) found within the centrum semiovale of the deep white matter. WML have been suggested to be ischaemic in origin, a component of SVD (9), and have been associated with the expression of hypoxia‐related cell pathways (10, 11). However, other mechanisms may contribute to WML including a loss of blood–brain barrier (BBB) integrity (12, 13), axonal damage secondary to cortical neuronal loss seen in AD (14), cerebral hypoperfusion (10) and potential ageing mechanisms such as DNA damage and cellular senescence (15, 16).

Microglia are the resident immune cell in the central nervous system (CNS) and have a major role in the immune response by exhibiting macrophage‐like actions, including the production of cytokines and chemokines (17), while astrocytes play a key role in maintaining brain homoeostasis and supporting neurons (18). Previous work using tissue from a longitudinal, population‐based study on ageing, the Cognitive Function and Ageing Study (CFAS) (19) that used post‐mortem MRI as a means of mapping WML (20), showed that WML have differing glial responses depending on whether they are DSCL or PVL (11, 21, 22). Clasmatodendritic (CD) astrocytes, immunoreactive for fibrinogen, were a prominent feature of WML, particularly PVL, suggesting BBB dysfunction contributes to their pathogenesis (11, 21). An increase in ramified, activated MHC‐II^+^ microglia in PVL also suggests that immune activation results from disruption of the BBB, while the presence of amoeboid phagocytic CD68^+^ microglia are a feature of DSCL (22). Furthermore, studies in CFAS have shown that WML are present in a more widespread field‐effect of abnormal white matter. The radiological normal appearing white matter areas from cases with WML show elevation of microglial markers, oxidative DNA damage and cell pathway alterations that are similar to those seen in WML, but are histologically different from control white matter from brains without lesions (11, 15, 22).

The current study defined the variation in the cellular (microglial and astrocytic) neuroinflammatory response in ageing white matter and to determine how patterns of inflammation relate to two potential drivers of white matter pathology, namely Alzheimer’s neuropathological change and SVD. We examined the detailed glial pathology of a representative subcohort from CFAS, an ageing population‐representative cohort that enables neuropathology investigations with an epidemiological approach (5, 10, 19, 23, 24). This approach allowed us to investigate the variation in neuropathology with ageing, and relationships to other pathologies, without preselection into clinical groups, which may introduce bias. We focused on changes in white matter in the cohort without preselecting for lesions, thus treating changes in white matter as a continuum. Using this approach, we interrogated the heterogeneity in patterns of the white matter neuroinflammatory response and examined the association of these patterns with AD and SVD neuropathological changes.

## 2 MATERIALS AND METHODS

### 2.1 Tissue

Human autopsy brain tissue was obtained from a single centre (Cambridge) of the CFAS cohort (n = 97), without preselection to maintain an unbiased population representative sample (25). Neuropathological lesions of these cases were assessed as part of the core CFAS neuropathology study using a modified protocol from the Consortium to Establish a Registry of Alzheimer’s Disease (CERAD), assessing burdens of plaques and tangles in multiple brain areas as mild, moderate or severe (26), and Braak neurofibrillary tangle (NFT) staging (27). Thal stage, cerebral amyloid angiopathy (CAA) and cortical and total microinfarcts were previously assessed in these cases (28, 29). Percentage area immunoreactivity of beta‐amyloid (Aβ) and tau pathology was assessed in parietal cortex as a further measure of local AD neuropathology (see below). Using a modified Scheltens score the extent of WML was determined by two independent radiologists using post‐mortem MRI of three anatomically defined coronal slices (20). Based on the size and number of WML, PVL were scored from 0 to 3, and DSCL from 0 to 6. The mean age of death was 86 years (95% CI = 84–87 years). The median post‐mortem delay was 17 h (Interquartile range (IQR) 7.5–27 h) and median brain pH 6.50 (IQR 6.24–6.74). This study examined the PARWM sampled from 97 cases of the Cambridge cohort, where 60 were female. Full case details are provided in Table 1. Research ethics committee (REC) approval was obtained for the study. (REC Reference number 15/SW/0246). The PARWM was selected as the parietal lobe is frequently shown to be involved in mild cognitive impairment through to clinically diagnosed AD (14, 30, 31, 32). Additionally, this area contains a large amount of white matter for analysis.

**TABLE 1:** Demographic details for the CFAS neuropathology cohort cases included in this study

Sample demographics	Men, n = 37 (38.14%)		Women, n = 60 (61.86%)		Total, n = 97 (100 %)	
Age at death, years (%)						
70–74	5	13.51	5	8.33	10	10.31
75–79	5	13.51	5	8.33	10	10.31
80–84	9	24.32	9	15.00	18	18.56
85–89	10	27.03	18	30.00	28	28.87
90+	8	21.62	23	38.33	31	31.96
Age at death, mean (95% CI)	83	(81–86)	83	(86–89)	86	(84–87)
Median post‐mortem interval, h (IQR)	21	(8–38)	15	(7–25.5)	17	(7.5–27)
Median brain PH (IQR)	6.43	(6.20–6.58)	6.54	(6.26–6.80)	6.50	(6.24–6.74)
Years of education, n (%)						
<10	25	71.43	37	67.27	62	68.89
10	3	8.57	8	14.55	11	12.22
>10	7	20.00	10	18.18	17	18.89
Median years of education (IQR)	9	(9–10)	9	(9–10)	9	(9–10)
Dementia Status, n (%)						
No dementia	16	45.71	21	35.00	37	38.95
Dementia	19	54.29	39	65.00	58	61.05
Diabetes mellitus, n (%)						
No diabetes	28	77.78	52	89.66	80	85.11
Diabetes	8	22.22	6	10.34	14	14.89
Hypertension, n (%)						
No hypertension	24	66.67	34	59.65	58	62.37
Hypertension	12	33.33	23	40.35	35	37.63

### 2.2 Immunohistochemistry

Immunohistochemistry was performed using a standard horseradish peroxidase‐conjugated avidin‐biotin complex (ABC‐HRP) method and the signal visualised with diaminobenzidine (DAB) as the chromogen (Vector Laboratories, Peterborough, UK). Sections were deparaffinised, rehydrated to water and endogenous peroxidase activity quenched by placing the sections in 0.3% H_2_O_2_/methanol for 20 min at room temperature (RT). Sections were subjected to antigen retrieval and following incubation with 1.5% normal serum for 30 min at RT, the sections were incubated with primary antibody, a summary of the primary antibodies and corresponding antigen retrieval methods and conditions used is shown in Table 2. Sections were washed thoroughly in tris‐buffered saline (TBS) and incubated with 0.5% of the relevant biotinylated secondary antibody (Vector Laboratories) for 30 min at RT. After washing in TBS the sections were incubated in ABC solution for 30 min at RT, followed by colour development with DAB. Negative controls were included in every run generated by either the omission of the primary antibody or an isotype control.

**TABLE 2:** Specificity, optimal dilution, antigen retrieval methods and source of antibodies used for immunohistochemistry

Antibody	Isotype	Dilution (time, temp)	Antigen retrieval method	Supplier
CD68	Mouse IgG 3	1:100 (1 h, RT)	TSC, pH6, PC	Abcam, PG‐M1, ab783
IBA1	Mouse IgG1κ	1:100 (1 h, RT)	TSC, pH6, PC	Millipore, MABN92
MHC‐II	Mouse IgG1κ	1:100 (1 h, RT)	TSC, pH6, PC	Dako, M0746
GFAP	Rabbit IgG	1:2000 (1 h, RT)	TSC, pH6, PC	Dako, Z0334
Collagen IV (COL4)	Rabbit IgG	1:500 (1 h, RT)	TSC, pH6, PC	Abcam, ab6586
Βeta‐Amyloid (Aβ)	Mouse IgG1κ	1:100 (o/n, 4°C)	^a^TSC, pH6, MW 10 min	Dako, M0872
Tau (AT8)	Mouse IgG_1_	1:400 (o/n, 4°C)	TSC, pH6, MW 10 min	Endogen MN1020

Abbreviations: GFAP, glial fibrillary acidic protein; IBA1, ionised calcium‐binding adaptor molecule 1; MHC‐II, major histocompatibility complex II; MW, microwave; PC, pressure cooker; RT, room temperature; TSC, tri‐sodium citrate buffer.

^a^ Following antigen retrieval sections stained for Aβ underwent pre‐treatment with formic acid for 60 min at RT.

### 2.3 Image analysis

Assessment of specific glial immunoreactivity was performed by capturing Brightfield microscopic images across a continuous non‐overlapping belt starting at the subventricular surface through to the subcortical area to cover the whole PARWM, using a x20 objective (Nikon Eclipse Ni‐U microscope, Nikon, UK) and analysed using the Analysis ^D software (Olympus Biosystems, Watford, UK). The belt area was chosen to include the largest amount of white matter on balance with integrity of the ventricular surface. Stellate and CD astrocytes were semi‐quantified in the three areas of white matter as none (0), frequent, up to 50% of astrocytes (1) very frequent more than 50% of astrocytes (3). For the sclerotic index (SI) score, Collagen IV (COL4) immunostained sections were digitally scanned under a 40x objective lens using a Nanozoomer XR (Hamamatsu, Photonics Ltd., Hertfordshire, UK). The final resolution of these images was 0.23 µm/pixel. Scanned sections were stored as NanoZoomer Digital Pathology Image (.ndpi) files, and viewed using NDP.View 2. Assessment of Aβ and tau immunoreactivity was performed by capturing Brightfield microscopic images across two continuous non‐overlapping belts starting from the outer cortex towards the inner cortex, stopping just before the white matter, using a x20 objective (Nikon Eclipse Ni‐U microscope, Nikon, UK) and analysed using the Analysis ^D software (Olympus Biosystems, Watford, UK.)

### 2.4 Assessment of small vessel pathology

**FIGURE 1:**
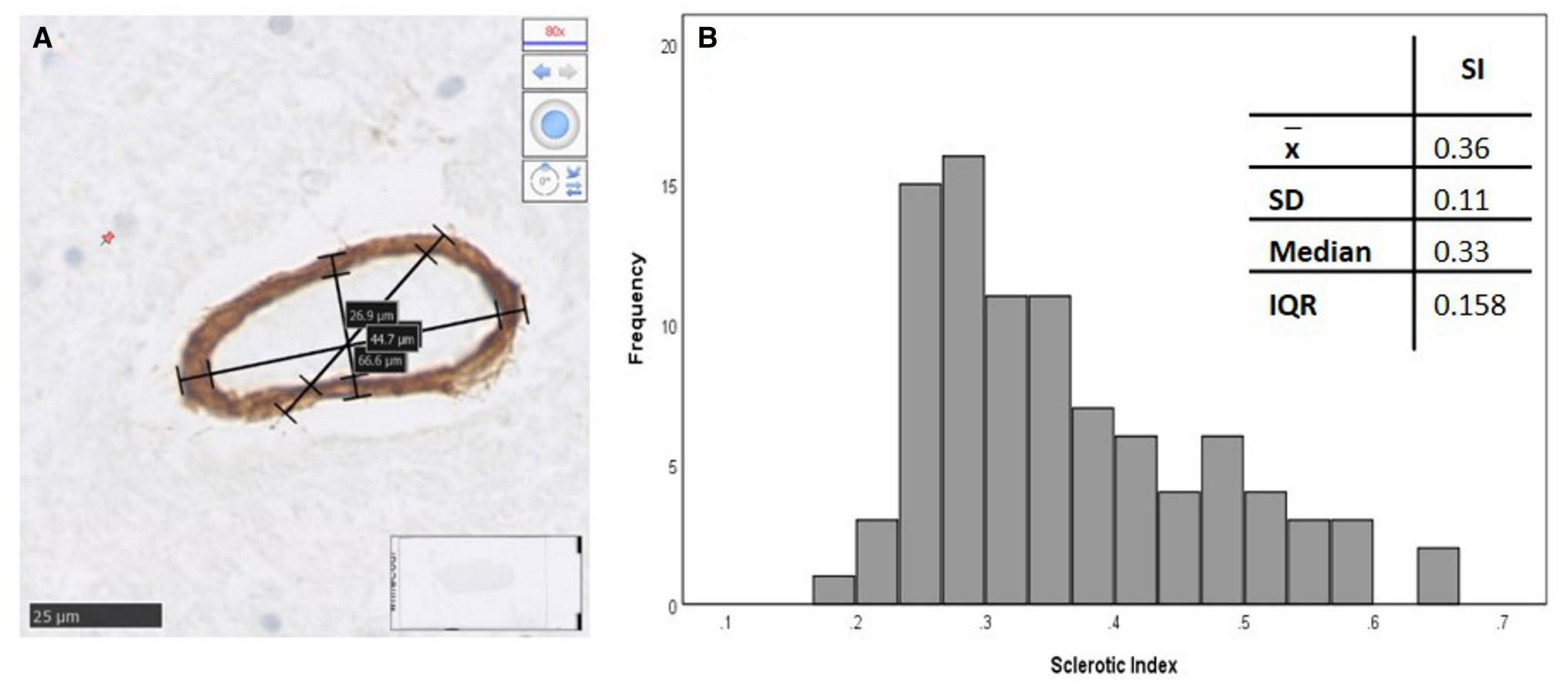
**Sclerotic Index (SI) calculated from collagen IV stained parietal section.** A small white matter artery, histologically stained with collagen IV, the internal and external diameters were measure three times to produce a SI value (A). The SI assessment across all cases in the cohort showed a positively skewed distribution (B)

To determine small vessel pathology within these cases, both the cortical and total microinfarct assessments were previously determined (28). Additionally, the total number of areas with CAA out of nine maximum was also determined (28, 29). The SI score allows the quantification of arteriolosclerosis as a measure of SVD severity (33, 34). The SI of each PARWM case used in this study was measured using COL4 immunostained scanned sections using the formula; SI = 1 − [Internal diameter (Dint)/External diameter (Dext)] (14). Using NDP.View 2 at x20 magnification, within the middle‐zone white matter region of each case, eight random arteries and/or arterioles >50 μm diameter were identified and the SI was calculated for each vessel (Figure 1A). Individual SI scores for each vessel were used to calculate an overall mean SI for each case. Assessment of the SI in the cohort was made by two independent individuals (JB and HH). To ensure consistency, both individuals assessed an overlap group of 10 cases. The mean difference between the two observers was 0.0018% (SD_d_ = 0.06), with no significant difference between their measurements (Wilcoxon signed‐rank test *p* = 0.683), showing consistent results between the two observers.

### 2.5 Assessment of AD neuropathological change

To determine AD neuropathological change in these cases, the global Braak and Braak staging for NFT was assessed previously by the analysis of AT8 (detects tau phosphorylated at serine 199/202) immunostained sections of hippocampus and isocortical regions. Thal staging had also been assessed previously in these cases by the analysis of Aβ immunostained sections of hippocampus and isocortical regions of tissue. Additional local assessment of AT8 and Aβ was made using percentage area of immunoreactivity in the cortex overlying the PARWM of these cases.

### 2.6 Statistical analysis

Statistical analyses were performed using IBM SPSS v24. Correlations between the white matter neuroinflammatory markers and proposed AD and SVD markers were assessed by Spearman’s Rank test. Differences in percentage area of GFAP between cases with and without thorn‐shaped astrocytes (TSA) were assessed by Mann–Whitney U test. Associations between scores for white matter astrocytes and TSA were assessed by Chi Square and the relationship between total number of areas with TSA and mean percentage area and subcortical GFAP immunoreactivity and with total white matter astrocyte scores was determined using Spearman’s Rank test. Diabetes mellitus and hypertension are dichotomised variables, therefore, logistic regression models were used to assess the associations between white matter neuroinflammatory markers, diabetes mellitus and hypertension. For assessment in relation to Braak NFT stages, cases were grouped into entorhinal stages (Group 1; Braak NFT stages I–II; 29 cases), limbic stage (Group 2; Braak NFT stages III–IV; 50 cases) and isocortical stages (Group 3; Braak NFT stages V–VI; 18 cases). Data were tested for normality using the Kolmogorov–Smirnov (KS) test. Variation of markers across the three areas of white matter was assessed using Friedman’s ANOVA (F) for related variables, and post hoc differences were assessed by Wilcoxon signed‐rank test, with *p*‐value corrected by the Bonferroni method. Interaction of variables was explored by principal components analysis (PCA). All tests were performed two tailed and significance set at *p* < 0.05.

## 3 RESULTS

### 3.1 Variation in microglial pathology across the white matter

**FIGURE 2:**
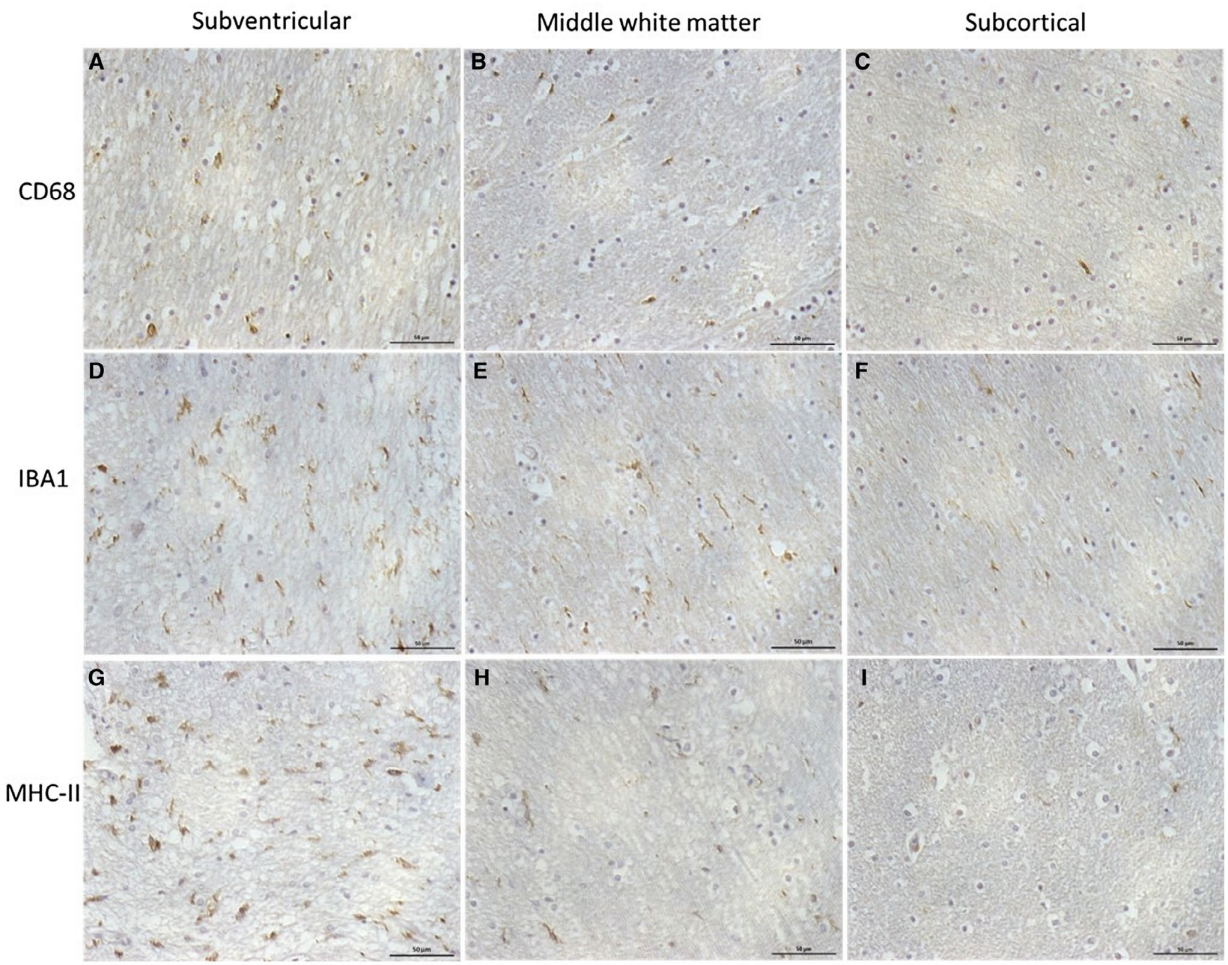
**Microglial immunoreactivity across the parietal white matter.** CD68, IBA1 and MHC‐II immunoreactivity in the subventricular (A, D, G), middle‐zone (B, E, H) and subcortical white matter regions (C, F, I). Scale bar represents 50 µm

**FIGURE 3:**
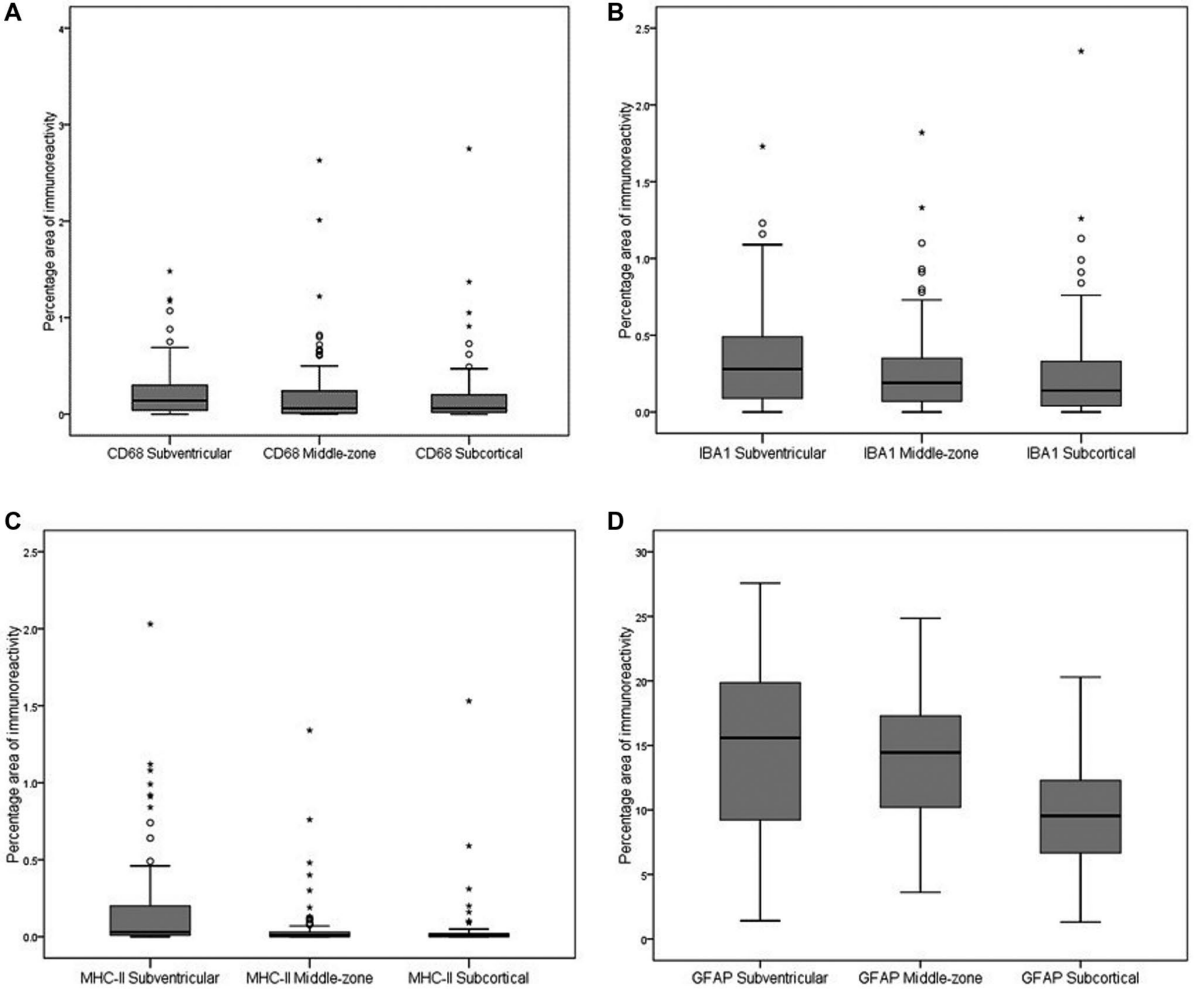
**Glia immunoreactivity in the subventricular, middle‐zone and subcortical parietal white matter regions.** CD68 (A), IBA1 (B), MHC‐II (C) and GFAP (D)

**FIGURE 4:**
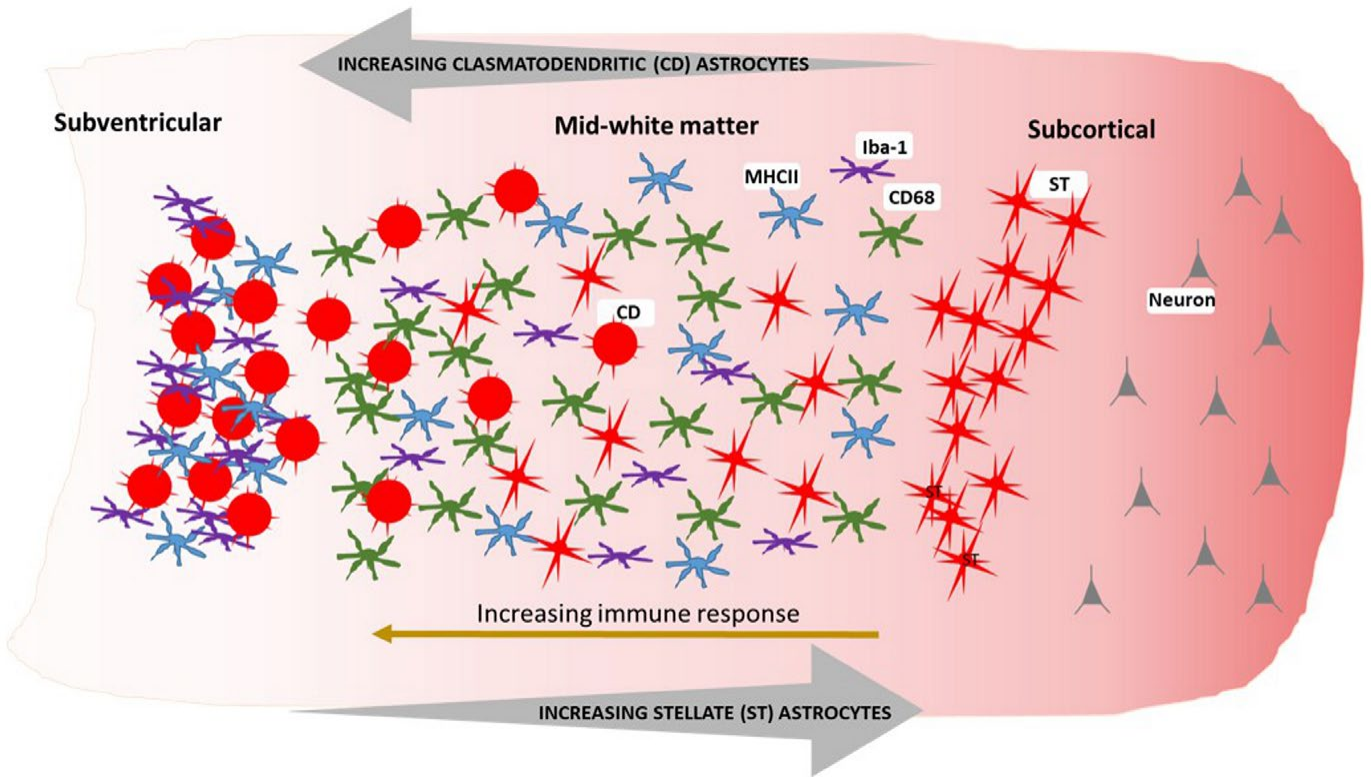
**Schematic representation of the pattern of the glial immune response throughout the ageing parietal white matter.** Microglial immunoreactivity is increased towards the subventricular region of the PARWM alongside an increasing presence of clasmatodendritic (CD) astrocytes. In contrast the presence of stellate (ST) astrocytes is increased towards the subcortical region of the PARWM

Immunohistochemical staining preparations of microglial markers CD68, IBA1 and MHC‐II were assessed as percentage area of immunoreactivity and as cell counts (Table 3). These two measures highly correlated for each of the three markers (all r_s_ > 0.9, *p* < 0.001). Therefore, for further analysis, percentage area of immunoreactivity was only used. The mean white matter expression of all three markers showed positively skewed distributions (KS *p* all < 0.001). The variation of microglial markers across the white matter was assessed by measuring the immunoreactivity in the subventricular, middle‐zone and subcortical areas of white matter (Figure 2, Table 4). CD68 significantly varied across the white matter (F = 10.94 2df *p* = 0.004) (Figure 3A). The subventricular zone had higher expression than either middle‐zone (*p* = 0.028) or subcortical region (*p* = 0.002), although only the latter remained significant after Bonferroni correction (*p* = 0.006). IBA1 showed variation across the white matter (F = 25.18 2df *p* < 0.001) (Figure 3B). Expression in the subventricular zone was higher than either middle‐zone (*p* < 0.001) or subcortical regions (*p* < 0.001). Although the subcortical region had lower values than the middle‐zone, this was not significant. MHC‐II expression also varied across the white matter (F = 49.40 2df *p* < 0.001) (Figure 3C), being higher in the subventricular region than either middle‐zone (*p* < 0.001) or subcortical zone (*p* < 0.001). The difference between mid and subcortical region was not significant after Bonferroni correction (*p* = 0.1). These microglial findings are summarised in Figure 4.

**TABLE 3:** Mean quantitation of microglial markers in parietal white matter

Marker	CD68 Cell count	CD68 % area	IBA1 Cell count	IBA1 % area	MHC‐II Cell count	MHC‐II % area
Mean	11.24	0.21	115.45	1.02	34.29	0.11
SD	14.82	0.32	88.66	4.18	91.20	0.37
Median	5.25	0.09	98.21	0.23	11.11	0.02
IQR	2.65–15.14	0.05–0.26	41.43–157.17	0.10–0.47	3.60–23.89	0.01–0.07
Correlation	r_s_ = 0.982, *p* ≤ 0.001		r_s_ = 0.938, *p* ≤ 0.001		r_s_ = 0.985, *p* ≤ 0.001	

Abbreviations: IQR, interquartile range; SD, standard deviation.

**TABLE 4:** Variation in percentage area of immunoreactivity of microglial markers across the parietal white matter [median (IQR)]

	Subventricular	Middle‐zone	Subcortical	Significance
CD68	0.14 (0.04–0.31)	0.06 (0.01–0.24)	0.02 (0.06–0.21)	*p* = 0.004
IBA1	0.28 (0.09–0.49)	0.19 (0.07–0.35)	0.04 (0.14–0.33)	*p* ≤ 0.001
MHC‐II	0.03 (0.01–0.21)	0.01 (0.00–0.04)	0.01 (0.00–0.02)	*p* ≤ 0.001

### 3.2 Variation in the astrocytic response across the white matter

**FIGURE 5:**
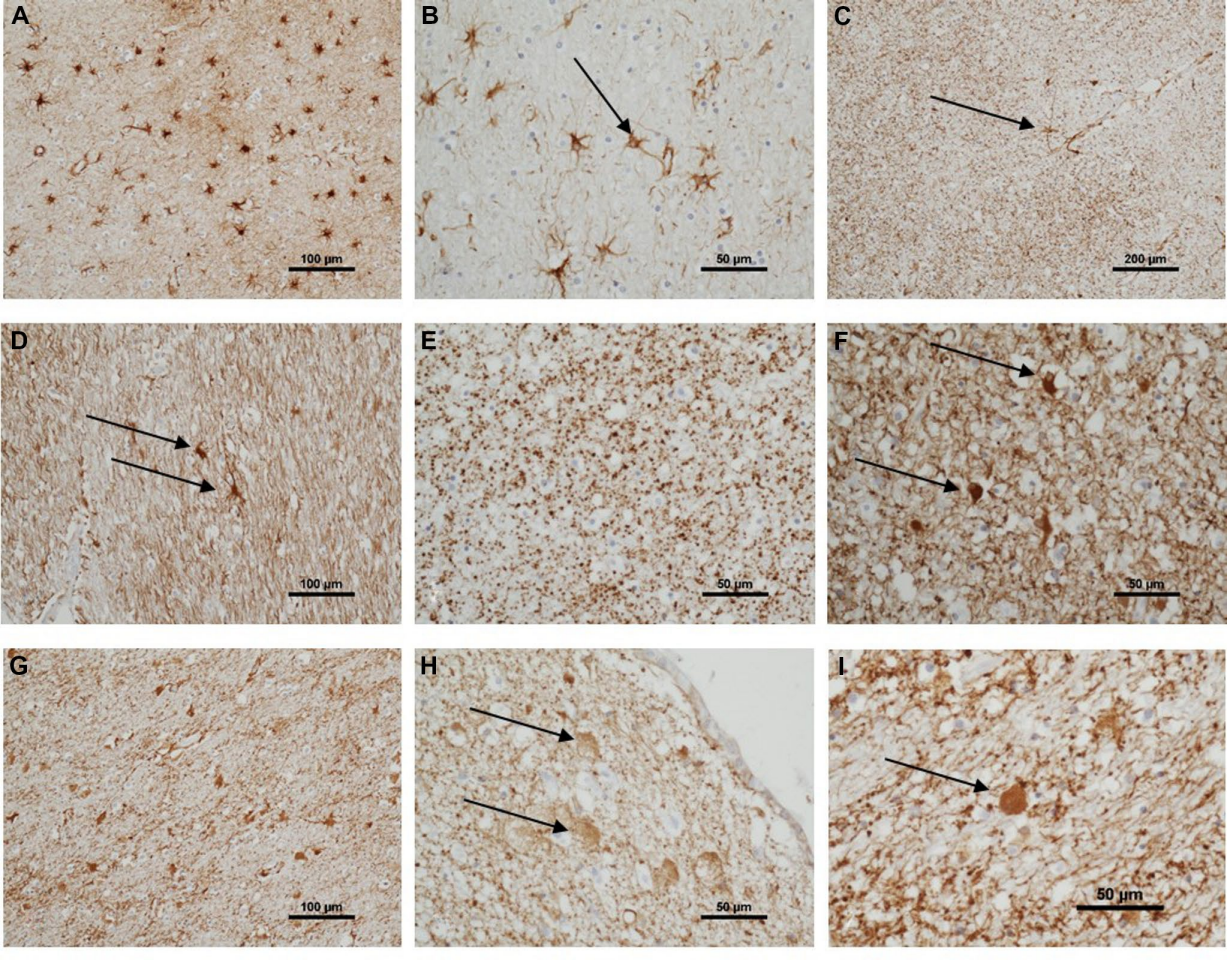
**Astrocytic pattern of immunoreactivity in parietal white matter.** A varied pattern of GFAP immunoreactivity across the parietal white matter. Frequent subcortical fibrillary astrocytes (A, [100 µm]). Fibrillary astrocytes detail, showing fine processes, one with a process to a vessel (B, arrow [50 µm]). Granular pattern of GFAP, an isolated fibrillary astrocyte has a process connecting to a capillary (C, arrow [200 µm]). Occasional fibrillary astrocytes in subventricular white matter, with a background of GFAP + glial processes (D, arrows [100 µm]). Granular GFAP pattern in middle‐zone white matter, without distinct astrocyte cell bodies (E, [50 µm]). Intermediate‐morphology astrocytes, with swollen GFAP + perikarya and short processes, in middle‐zone of white matter (F, arrows [50 µm]). Frequent clasmatodendritic astrocytes in middle‐zone white matter (G [100 µm]). Clasmatodendritic astrocytes in subcortical white matter (H, arrows [50 µm]). High power of clasmatodendritic astrocytes showing the swollen GFAP + body without processes (I, [50 µm])

**FIGURE 6:**
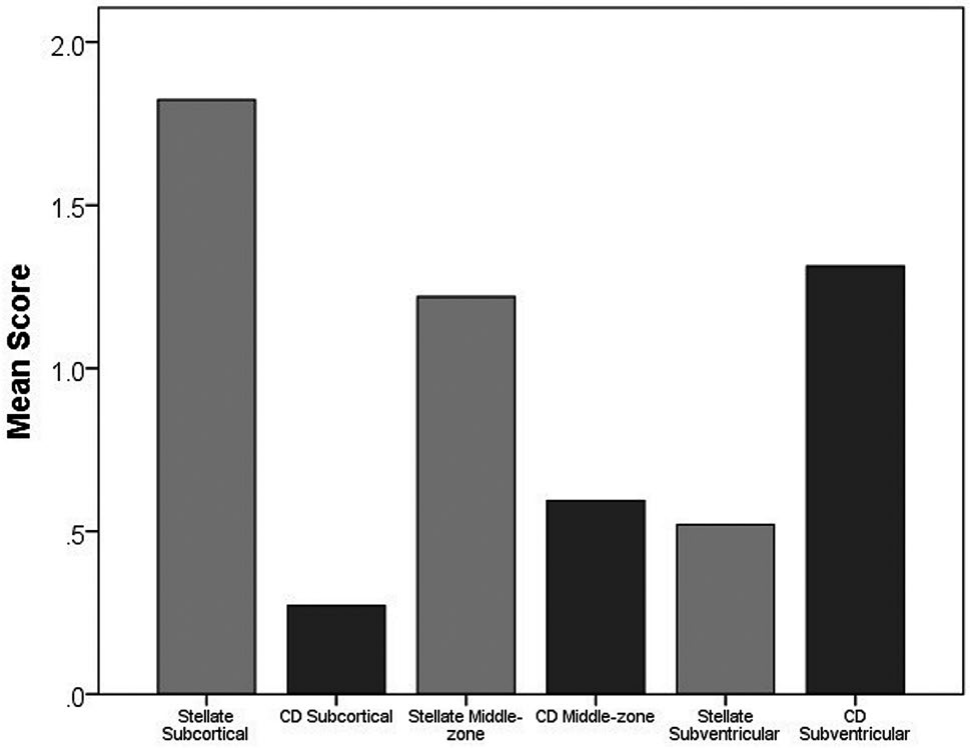
**Astrocyte morphology across the parietal white matter.** Stellate astrocytes decrease from subcortical through to subventricular regions, while clasmatodendritic astrocytes increase

Astrocytes were identified using immunohistochemistry to GFAP and quantified as percentage area of immunoreactivity (Table S1). The mean white matter GFAP expression was normally distributed (KS *p* = 0.2) (Figure 3D). GFAP expression showed variation across the white matter (F = 52.90 2df *p* < 0.001), with the subventricular zone having higher expression than either middle‐zone (*p* < 0.001) or subcortical (*p* < 0.001). GFAP^+^ astrocytes within white matter showed variation in morphology (Figure 5): some had a fibrillary or stellate appearance with processes (Figure 5A–D); others had a more rounded appearance without processes, a CD appearance (Figure 5G–I). When frequent, these were associated with a granular appearance to the background GFAP staining suggesting fragmentation of astrocyte processes (Figure 5E). Cells with an intermediate appearance were also noted (Figure 5F). We semi‐quantified the stellate and CD astrocytes in the three areas of white matter (subventricular, middle‐zone and subcortical). Scores for stellate astrocytes (F = 124.1 2df *p* < 0.001) and CD astrocytes (F = 93.8 2df *p* < 0.001) differed between the three regions of white matter: stellate astrocyte scores decreased from subcortical through to subventricular, whereas, CD astrocytes increased suggesting a gradient of white matter neuroinflammation and BBB leakage towards the subventricular zone (Figure 6). The overall total sum scores for stellate and CD astrocytes were negatively correlated (r_s_ = −0.75, *p* < 0.001), so that these morphologies were inversely related. These astrocyte findings are summarised in Figure 4.

We also assessed whether these astrocyte changes in white matter showed any relationship to another common age‐associate glial pathology, ageing‐related tau astrogliopathy (ARTAG), which may also affect the white matter. We, therefore, assessed the relationship to score for TSA, previously assessed on the cohort, which are a key feature of ARTAG (35). GFAP percentage area of immunoreactivity in the subcortical white matter was higher in cases with TSA in any area of the PARWM (Mann Whitney *p* = 0.041) but the overall mean percentage area of GFAP immunoreactivity and total stellate astrocyte score did not vary with TSA. The Score for subcortical astrocytes did not increase with TSA in any area of the PARWM (χ^2^ = 3.292, 2df, *p* = 0.193). The total number of areas with TSA showed no significant correlations with mean percentage area of GFAP immunoreactivity, subcortical percentage area of GFAP immunoreactivity or total stellate astrocyte score. The preliminary observation of elevated GFAP in the subcortical region in cases with ARTAG suggests a relationship to other forms of ageing brain pathology.

### 3.3 Expression of some neuroinflammatory markers relate to MRI white matter lesion score

Scheltens score for DSCL showed moderate correlation with CD68 (r_s_ = 0.48, *p* < 0.001), IBA1 (r_s_ = 0.32, *p* = 0.001) and GFAP (r_s_ = 0.30, *p* = 0.003). However, MHC‐II did not correlate with this MRI rating (r_s_ = 0.07, *p* = 0.48). Similar significant correlations were observed when just the DSCL score for the mid‐level slice was used. The score for PVL showed limited correlation with mean CD68 (r_s_ = 0.39, *p* < 0.001) and IBA1 (r_s_ = 0.23, *p* = 0.030) but not GFAP (r_s_ = 0.15, *p* = 0.144). There was only a weak correlation between PVL score and MHC‐II that did not reach significance (r_s_ = 0.20, *p* = 0.059), but there was a limited correlation when just the PVL score for the mid‐level slice was used alone (r_s_ = 0.25, *p* = 0.018). To further explore the relationship of these white matter neuroinflammatory markers to imaging in the periventricular region, the immunoreactivity specifically of the glial markers in the subventricular region was considered. However, none of these associations reached significance (data not presented). Neither stellate astrocyte score, nor CD astrocytes showed correlation with MRI scores for DSCL or PVL.

### 3.4 Relationships between neuroinflammatory markers suggest different patterns

These results suggested differences between the neuroinflammatory markers across the breadth of the PARWM and this may reflect different drivers of white matter pathology. We, therefore, examined the relationship between the overall expression of glial markers in the PARWM and lesional score of the cases to identify those relationships with significant correlations with effect size, r_s_, of 0.25 or greater (Table 5). The positive correlations (Table 5, red) between the overall GFAP expression, CD astrocytes and microglial markers suggested a relationship between all these neuroinflammatory markers. However, whilst CD astrocytes positively correlated with IBA1 and MHC‐II expression, they did not correlate with CD68 expression in the cases. Additionally, stellate astrocytes did not correlate with CD68 whilst showing only limited negative correlations (Table 5, green) with IBA1 and MHC‐II. CD68 and IBA1 showed the strongest correlations with MRI‐defined WML, whereas CD astrocytes, stellate astrocytes and MHC‐II did not.

**TABLE 5:** Correlation matrix r_s_, *p* (red positive correlation [r_s_ ≥ 0.25 and *p* < 0.05], green negative)

Variables	GFAP	Stellate astrocytes	CD astrocytes	CD68	IBA1	MHC‐II	MRI DSCL^a^	MRI PVL^a^
GFAP		−0.35, 0.001	0.29, 0.004	0.25, 0.013	0.45, <0.001	0.36, <0.001	0.30, 0.003	0.15, 0.144
Stellate astrocytes			−0.75, <0.001	0.01, 0.92	−0.21, 0.037	−0.21, 0.042	0.01, 0.930	0.02, 0.858
CD astrocytes				0.05, 0.63	0.29, 0.005	0.27, 0.008	0.05, 0.619	0.01, 0.895
CD68					0.46, <0.001	0.16, 0.12	0.48, <0.001	0.39, <0.001
IBA1						0.25, 0.012	0.32, 0.001	0.23, 0.030
MHC‐II							0.07, 0.479	0.20, 0.059
MRI DSCL^a^								0.67, <0.001
MRI PVL^a^								

^a^ MRI score for mid slice shows the same significant correlations as the total DSCL/PVL score.

**FIGURE 7:**
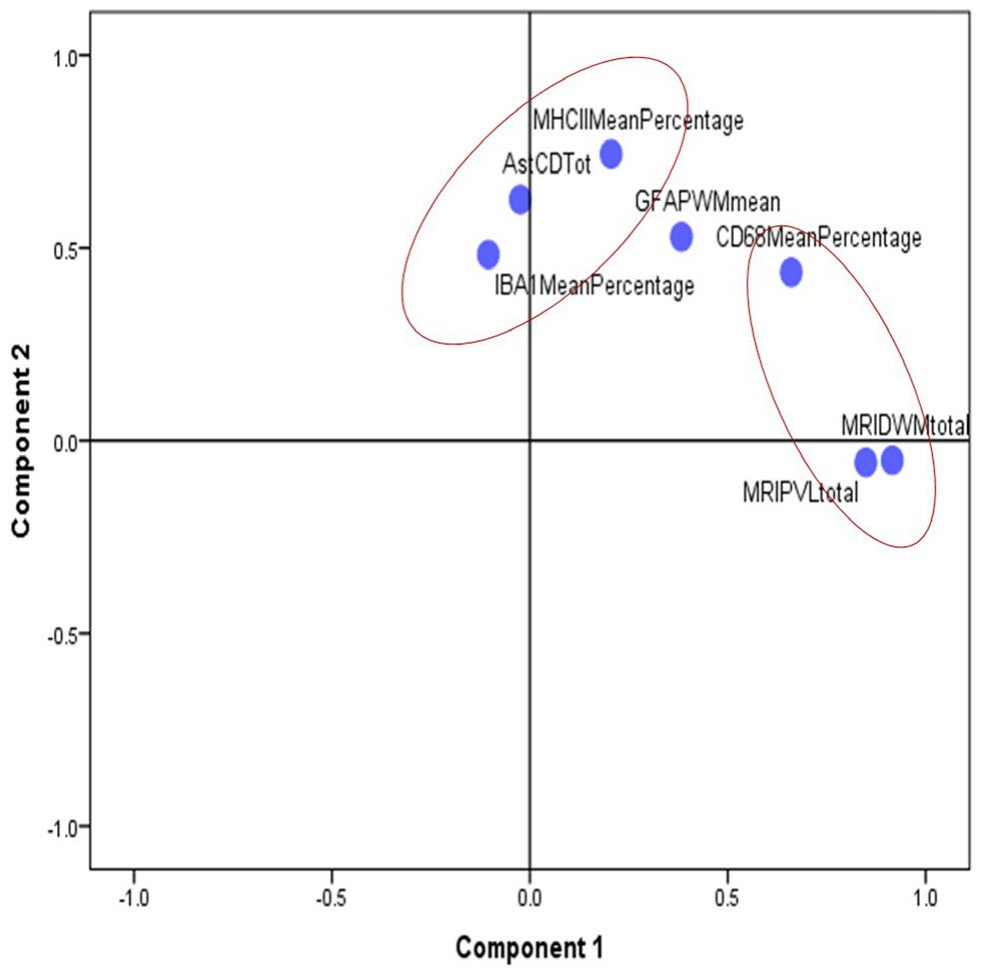
**Principal components analysis (PCA) plot of neuroinflammatory marker interactions.** PCA identified two principal components (PC) that explained approximately 55% of the variance in the selected variables in the white matter, with the MRI DSCL and PVL lesion scores and CD68 (lesional pattern 2) most closely aligned to component 1, and CD astrocytes, IBA1 and MHC‐II (immune pattern 1) most closely aligned to component 2

The interactions between GFAP, CD astrocytes, CD68, IBAI, MHC‐II, MRI DSCL and PVL were further visualised using principal components analysis (PCA) (Figure 7). PCA identified two principal components (PC) that explained approximately 55% of the variance in the selected variables in the white matter. MRI DSCL, MRI PVL and mean percentage of CD68 immunoreactivity (lesion pattern 2) contributed most to PC1, whilst CD astrocytes, IBA1 and MHC‐II immunoreactivity (immune pattern 1) contributed most to PC2. This suggests there is more than one pattern of white matter injury, although with some overlap in cellular responses. We, therefore, defined two patterns for further analysis: Pattern 1– an immune pattern characterised by the presence of CD astrocytes and elevated MHC‐II expression and Pattern 2– a lesional pattern, with identifiable WML on MRI, associated with increases in CD68^+^ microglial but not CD astrocytes or MHC‐II. GFAP expression increased with all of the microglial markers and seemed to be less specific.

### 3.5 Relationship to vascular and AD neuropathology

We next assessed how these different patterns of white matter cellular pathology related to potential causes of age‐associated white matter injury.

#### 3.5.1 No relationship to Alzheimer’s neuropathology

We then assessed the relationships to Braak NFT stage and Thal Aβ stage as measures of global brain Alzheimer’s neuropathological changes and to the parietal cortex percentage area of immunoreactivity for tau and Aβ as local measures of AD neuropathology in cortex overlying the PARWM. None of the white matter neuroinflammatory markers showed a relationship to these variables (see Table S2), apart from IBA1 which showed a weak correlation with local parietal percentage area immunoreactivity of tau (r_s_ = 0.229, *p* = 0.039).

#### 3.5.2 Weak relationships to markers of microvascular pathology

To determine how white matter cellular inflammatory responses relate to potential vascular drivers, we examined the relationships to several markers of SVD, including cortical microinfarct stage, number of areas with CAA and the SI. SI, a marker of arteriolosclerosis showed a positively skewed distribution (KS *p* all < 0.001, Figure 1B) with cases having an average SI of 0.36, typical of mild SVD.

Pattern 1: CD astrocytes showed a limited correlation with the number of areas with CAA (r_s_ = 0.22, *p* = 0.031), but not with cortical microinfarct stage (r_s_ = 0.02, *p* = 0.832), total number of microinfarcts (r_s_ = 0.02, *p* = 0.878) or SI (r_s_ = 0.07, *p* = 0.488). MHC‐II showed weak correlations with cortical microinfarct stage (r_s_ = 0.26, *p* = 0.011), total microinfarcts (r_s_ = 0.23, *p* = 0.023) and SI (r_s_ = 0.27, *p* = 0.010) but not with number of areas with CAA (r_s_ = 0.13, *p* = 0.219). IBA1 correlated weakly with SI (r_s_ = 0.27, *p* = 0.010) but not with the number of areas with CAA (r_s_ = −0.07, *p* = 0.409), cortical microinfarct stage (r_s_ = 0.09, *p* = 0.407) or total number of microinfarcts (r_s_ = 0.17, *p* = 0.104).

Pattern 2: MRI DSCL showed no correlation to CAA (r_s_ = 0.14 *p* = 0.17), cortical microinfarcts (r_s_ = 0.16 *p* = 0.13) or total microinfarcts (r_s_ = 0.19 *p* = 0.07). MRI PVL showed no correlation to CAA (r_s_ = 0.18 *p* = 0.08), cortical microinfarcts (r_s_ = 0.18 *p* = 0.09) or total microinfarcts (r_s_ = 0.13 *p* = 0.23). CD68 showed no correlation to CAA (r_s_ = −0.01 *p* = 0.92), cortical microinfarcts (r_s_ = 0.01 *p* = 0.89) or total microinfarcts (r_s_ = 0.08 *p* = 0.46). SI showed marginal, weak correlations to CD68 (r_s_ = 0.21, *p* = 0.047), MRI diffuse score (r_s_ = 0.21, *p* = 0.049), total MRI PVL score (r_s_ = 0.25, *p* = 0.022) but not to total MRI DSCL score (r_s_ = 0.14, *p* = 0.202).

GFAP percentage area of immunoreactivity did not correlate with vascular measures including cortical (r_s_ = 0.09, *p* = 0.403), or total microinfarct stage (r_s_ = 0.11, *p* = 0.280), number of areas with CAA (r_s_ = −0.02, *p* = 0.851), and the SI (r_s_ = 0.16, *p* = 0.141) of this cohort.

### 3.6 No relationship to diabetes mellitus or hypertension

We assessed whether there was any relationship between white matter cellular changes and other comorbidities in the cases including diabetes mellitus and hypertension. None of the white matter neuroinflammatory markers showed a relationship to diabetes mellitus or hypertension status (see Table S3), apart from total score of stellate astrocytes which showed only a weak negative correlation with hypertension (Coef = 0.326, *p* = 0.023). However, caused by the small number of cases with these co‐morbidities, this analysis was inconclusive.

## 4 DISCUSSION

In the current study, we defined the neuroinflammatory response in the cerebral white matter of an unselected, population‐derived brain‐donation cohort. This response is heterogeneous between individuals and varies across the PARWM from the subcortical to subventricular region. We show that: i. Expression of microglial markers (CD68, IBA1 and MHC‐II) and the astrocytic marker (GFAP) increase from the subcortical to the subventricular white matter, ii. Astrocytes with a stellate morphology decrease from the subcortical to subventricular region, whilst CD astrocytes increase, iii. WML defined by MRI correlate with CD68 expression, whilst CD astrocytes and MHC‐II expression are related suggesting that there are different patterns of white matter neuroinflammation, a lesional pattern with innate microglial reactivity and a more immune pattern and iv. White matter neuroinflammation shows only weak relationships to markers of microvascular pathology and no relationship to markers of Alzheimer’s neuropathology.

The activation of microglia and astrocytes is a feature of neurodegenerative diseases (36, 37, 38). The current study demonstrates that neuroinflammation in the PARWM of the ageing brain is a complex phenomenon that is driven by several entities. Variation in the glial phenotype across WML subtypes defined by their anatomical location has already been reported, with significantly more CD68^+^ microglia present in DSCL (22, 39), MHC‐II^+^ microglia in PVL (22) and CD astrocytes, associated with BBB leakage, a prominent feature of PVL (21). CD astrocytes in the frontal white matter of post‐stroke demented (PSD) subjects occur at higher frequencies compared to post‐stroke individuals without dementia (PSND), with a loss of aquaporin 4 (AQP4) and white matter pericytes, suggesting that changes in astrocytic function and disruption of the gliovascular unit at the BBB contribute to dementia after stroke (40, 41). The increased number of CD astrocytes and the elevated expression of all microglial markers in the subventricular zone is suggestive of BBB changes in this region and not restricted to areas containing WML.

Correlation analysis of this ageing population‐representative cohort identified grouping of specific microglia and astroglial markers, suggesting that there are heterogeneous patterns of neuroinflammation throughout the white matter. The grouping of CD astrocytes with MHCII^+^ and IBA1^+^ microglia suggests an immune pattern, which reflects disruption of the BBB leading to serum protein deposition exacerbating microglial activation (40, 42). This is compared to a more lesional pattern with innate CD68^+^ microglial reactivity associated with elevated MRI scores for WML.

The causes of white matter changes in ageing is debatable and, to‐date, studies have not taken into account the heterogeneity of the cellular changes in white matter. Alzheimer type pathology is one candidate for causing secondary white matter damage. Previous work has shown that WML in cases diagnosed as having AD neuropathology are associated with Wallerian degeneration as a consequence of cortical AD‐pathology including hyperphosphorylated tau and Aβ (14, 43, 44). However, these studies, as well as others, acknowledge that mixed pathologies exist, with vascular changes occurring in AD cases and vice versa (5, 45, 46). Therefore, determining whether the pathological and molecular signatures differ between those individuals with WML associated with cortical AD pathology compared to those individuals with WML associated with SVD is warranted. Other factors must also be considered when looking at overall white matter changes, for example, AD is often associated with increased CAA deposition which can cause vascular effects on the white matter (45, 47, 48, 49). Additionally, hypoperfusion, including evidence of hypoxic‐induced alterations in protein expression in areas of the brain with Aβ pathology may contribute to AD (50, 51). Clearly, separating and classifying these confounding mechanisms responsible for white matter changes are difficult. We fail to show any correlation of either pattern of white matter neuroinflammatory response to markers of AD in this population‐representative cohort suggesting that the white matter neuroinflammatory changes is not related to the tau and Aβ component of AD.

An alternative potential driver of white matter neuroinflammation is vascular pathology, an important contributor to dementia (52). CFAS has shown that white matter vascular pathology is frequent, being observed in 71% of non‐demented individuals versus 84% of demented individuals, and with 60% of non‐demented individuals exhibiting evidence of SVD. However, this study did not correlate the extent of WML and SVD in the patient cohort (5). The current study showed associations between measures of vascular pathology, particularly SI, and both patterns of white matter neuroinflammation, suggesting that there is an effect of vascular pathology on white matter neuroinflammation in contrast to the lack of effect with AD. However, although statistically significant the effect sizes as measured by correlation coefficient showed that the associations between vascular pathology and white matter neuroinflammation are weak.

Although AD and SVD have, respectively, been considered to be important drivers of WML and the field‐effect of diffuse abnormal white matter, the lack of strong associations in this study suggests that other factors may contribute to white matter damage. Ageing, a major risk factor for WML, could operate though several cellular mechanisms, and these remain to be investigated. We examined the relationship of PARWM astroglial changes to another important and recently defined age‐related pathology of astrocytes, ARTAG, which may involve white matter (53, 54). Subcortical stellate astrocytes and subcortical GFAP reactivity both increased in the presence of TSA, a feature of ARTAG, in any brain area. We did not examine whether phospho‐tau was expressed in subcortical astrocytes but this preliminary observation of an association between ARTAG and subcortical astrocyte reactivity suggests a relationship of white matter glial changes to other forms of ageing brain pathology.

Disruption the neurovascular unit is also a candidate factor. Disruption of the BBB and the resulting influx of neurotoxic blood‐derived products, cells and pathogens may promote white matter neuroinflammation and impact neuronal function. This has been previously identified in the ageing white matter as evidenced by the accumulation of serum protein in CD astrocytes (21), but BBB disruption has also been identified in many other disorders including multiple sclerosis (55), amyotrophic lateral sclerosis (56), chronic traumatic encephalopathy (57), Parkinson’s Disease (58) and Huntington’s Disease (59). A more thorough investigation at the cellular level of the gliovascular unit in the white matter is warranted, including the endothelial cells and pericytes along with the tight junction proteins that maintain the BBB integrity. Recent work has shown a 35%–44% decrease in capillary pericytes in the frontal lobe across different dementias including PSD, vascular dementia, mixed and AD suggesting that the reduction in pericytes contributes to BBB disruption in the white matter (41). It would be interesting to see whether the reduction in pericytes is seen more widespread throughout different brain regions, including the PARWM investigated in this study.

Ageing may also contribute to white matter pathology through other processes such as mitochondrial dysfunction, cellular senescence, disturbances in intracellular communication and genomic instability (60). Oxidative glial damage is associated with WML in the ageing brain (61). Also, as neuroinflammation and gliosis increase with age, profound gene expression changes across the whole brain are prominent, with transcriptomic analysis revealing higher inflammatory profiles associated with increased age (62). Another confounding ageing process affecting white matter is venous collagenosis which induces ischaemia, increasing vascular resistance and fluid leakage causing oedema and hypoperfusion associated with white matter pathology (63, 64). In addition to AD pathology and SVD, the multiple cellular processes associated with ageing are factors that also need to be considered when assessing the potential causes of white matter changes.

### 4.1 Study limitations

This study investigated a restricted panel of markers in the white matter of one brain region, the PARWM. As seen from other studies the neuroinflammatory response differs based on age and neuroanatomical location (65, 66). Also the CD astrocyte response in PSD was limited to the frontal white matter and not seen in the temporal white matter (40). Furthermore, we have not defined white matter neuroinflammation at a molecular level, solely examining the expression of astrocytic and microglial markers which is a limited approach when assessing glial responses and their involvement in white matter neuroinflammation. Additionally, we did not define the subtypes of microglial response or distinguish the overall function of the glia which may give a better indication of their inflammatory subtype (67). Despite an immune‐related pattern of white matter neuroinflammation being associated with the subventricular area of the PARWM, the overall SI scores assessed in this patient cohort were taken from the middle‐zone of the PARWM, not including the extremities of the subventricular and subcortical areas. Classical markers of AD including immunostaining for hyperphosphorylated tau and Aβ, which form the Braak and Thal staging of AD, respectively, failed to show association with white matter neuroinflammation in this cohort. However, this study does not take Aβ plaque subtypes into account and future studies investigating the association with oligomeric Aβ, for example, are required. Furthermore, association with markers of neuronal damage should be assessed, as white matter neuroinflammation may impact axonal function leading the neuronal damage. whilst the current study investigated a cohort of approximately 100 cases, given the heterogeneity of the white matter neuroinflammatory response, a major limitation is the number of cases examined. This is clearly apparent when investigating the potential relationship between white matter neuroinflammatory responses and other comorbidities within the cases; including diabetes mellitus and hypertension. No significant findings were found, possibly caused by the small number of cases with comorbidities in the cohort. Therefore, increasing the cohort size and extending the panel of markers investigated may uncover relationships between AD or SVD pathology and neuroinflammation in the ageing white matter.

### 4.2 Conclusions

In summary, our findings have shown distinct cellular patterns of neuroinflammation in the PARWM of the ageing brain. Recognising and further defining this heterogeneity and its significance in a larger ageing cohort are important to fully elucidate the cause and impact of WML pathogenesis. Such an approach will be important in understanding the therapeutic targets for age‐associated white matter damage.

## CONFLICT OF INTEREST

The authors have no conflict of interest.

## AUTHOR CONTRIBUTIONS

Conception of the project; SW. Experimental work and data collection; RW, RN, NV, MT, JB, HH and FEH. Study design and supervision; SW, JS, PH and RW. Data analysis; SW and RW. Statistical advice; FM and CDR. Writing of first draft SW, RW and JS. CFAS data custodianship, provision of clinical/demographic data and epidemiological interpretation; FM, CB and CDR. Collation of author contributions into final draft; RW, SW and JS. Contribution to interpretation, literature context and to the final manuscript; all authors.

## ETHICAL APPROVAL

Human autopsy brain tissue used in this study was obtained from a single centre (Cambridge) of the CFAS cohort following Research Ethics Committee (REC) approval (REC Reference number 15/SW/0246).

## Supporting information

Table S1
**Table S1** Variation in GFAP immunoreactivity across the white matter. SD, standard deviation and IQR, interquartile range

Table S2
**Table S2** Alzheimer’s neuropathology correlation matrix. None of the neuroinflammatory markers correlated with markers of Alzheimer's pathology. *IBA1 expression negatively correlated with Local Tau immunoreactivity (r_s_, *p*‐value)

Table S3
**Table S3** Logistic regression analysis for diabetes and hypertension. Multivariate model was adjusted for age at death and sex. Results presented with correlation coefficients (Coef), 95% confidence intervals (95% CI) and *p*‐values (*p*)

## Data Availability

The data that support the findings of this study are available from the corresponding author upon reasonable request.
