# Supplementary material for: Heterogeneity of cellular inflammatory responses in ageing white matter and relationship to Alzheimer’s and small vessel disease pathologies
Source: Brain Pathol. 2021 Feb 15;31(3):e12928. doi: 10.1111/bpa.12928 (PMC8412112; doi:10.1111/bpa.12928)
Supplement: Supplementary file 1 — Table S1 Table S1 Variation in GFAP immunoreactivity across the white matter. SD, standard deviation and IQR, interquartile range [file BPA-31-e12928-s001.rtf]

Supplementary Table 1
% GFAP immunoreactivity 	Mean white matter	Subventricular	Middle-zone	Subcortical	
Mean (SD)	12.86 (3.95)	14.81 (6.51)	13.91 (4.98)	9.81 (4.11)	
Median (IQR)	13.20 (10.09-15.66)	15.59 (8.98-19.88)	14.46 (10.18-17.30)	9.54 (6.66-12.38)	
