# Supplementary material for: Heterogeneity of cellular inflammatory responses in ageing white matter and relationship to Alzheimer’s and small vessel disease pathologies
Source: Brain Pathol. 2021 Feb 15;31(3):e12928. doi: 10.1111/bpa.12928 (PMC8412112; doi:10.1111/bpa.12928)
Supplement: Supplementary file 2 — Table S2 Table S2 Alzheimer’s neuropathology correlation matrix. None of the neuroinflammatory markers correlated with markers of Alzheimer's pathology. *IBA1 expression negatively correlated with Local Tau immunoreactivity (rs, p‐value) [file BPA-31-e12928-s003.rtf]

Supplementary Table 2
Pattern 1	Local AB	Local Tau	Braak NFT	Thal a beta	
CD astrocytes	.13, 0.22	.12, 0.28	.05, 0.62	.05, 0.60	
MHC-II	.02, 0.86	-.18, 0.10	-.01, 0.94	.04, 0.67	
IBA1	-.08, 0.46	-.23, 0.03*	-.09, 0.36	-.08, 0.43	
GFAP	-.08, 0.46	-.18, 0.10	-.02, 0.84	-.07, 0.52	

Pattern 2	Local AB	Local Tau	Braak NFT	Thal a beta	
MRI DSCL	-.17, 0.10	-.18, 0.10	.14, 0.16	.05, 0.64	
MRI PVL	-.03, 0.78	-.12, 0.27	.15, 0.15	.06, 0.62	
CD68	-.07, 0.40	-.17, 0.13	-.06, 0.54	.02, 0.81	
