# Supplementary material for: Heterogeneity of cellular inflammatory responses in ageing white matter and relationship to Alzheimer’s and small vessel disease pathologies
Source: Brain Pathol. 2021 Feb 15;31(3):e12928. doi: 10.1111/bpa.12928 (PMC8412112; doi:10.1111/bpa.12928)
Supplement: Supplementary file 3 — Table S3 Table S3 Logistic regression analysis for diabetes and hypertension. Multivariate model was adjusted for age at death and sex. Results presented with correlation coefficients (Coef), 95% confidence intervals (95% CI) and p‐values (p) [file BPA-31-e12928-s002.docx]

| *Diabetes mellitus* | Unadjusted | | | | Adjusted | | | |
| --- | --- | --- | --- | --- | --- | --- | --- | --- |
|  | *Coef.* | *(95% CI)* | | *p=* | *Coef.* | *(95% CI)* | | *p=* |
| *GFAP* | *0.01* | *(-0.13,* | *0.16)* | *0.84* | *0.00* | *(-0.15,* | *0.16)* | *0.98* |
| *Stellate astrocytes* | *0.05* | *(-0.31,* | *0.41)* | *0.77* | *0.07* | *(-0.30,* | *0.45)* | *0.70* |
| *CD astrocytes* | *0.01* | *(-0.29,* | *0.30)* | *0.97* | *0.05* | *(-0.26,* | *0.36)* | *0.77* |
| *CD68* | *-0.75* | *(-3.35,* | *1.86)* | *0.58* | *-0.49* | *(-3.30,* | *2.31)* | *0.73* |
| *IBA1* | *0.00* | *(-0.13,* | *0.13)* | *0.97* | *0.03* | *(-0.11,* | *0.16)* | *0.72* |
| *MHC-II* | *-1.35* | *(-6.40,* | *3.69)* | *0.60* | *-0.69* | *(-5.36,* | *3.98)* | *0.77* |
| *Hypertension* | Unadjusted | | | | Adjusted | | | |
|  | *Coef.* | *(95% CI)* | | *p=* | *Coef.* | *(95% CI)* | | *p=* |
| *GFAP* | *0.06* | *(-0.05,* | *0.17)* | *0.26* | *0.06* | *(-0.05,* | *0.17)* | *0.32* |
| *Stellate astrocytes* | *-0.33* | *(-0.62,* | *-0.05)* | *0.02* | *-0.33* | *(-0.61,* | *-0.04)* | *0.02* |
| *CD astrocytes* | *0.07* | *(-0.15,* | *0.29)* | *0.55* | *0.07* | *(-0.15,* | *0.29)* | *0.54* |
| *CD68* | *0.20* | *(-1.32,* | *1.71)* | *0.80* | *0.12* | *(-1.41,* | *1.65)* | *0.88* |
| *IBA1* | *-0.03* | *(-0.15,* | *0.09)* | *0.61* | *-0.03* | *(-0.15,* | *0.09)* | *0.60* |
| *MHC-II* | *0.95* | *(-1.13,* | *3.02)* | *0.37* | *0.90* | *(-1.20,* | *2.99)* | *0.40* |

**Supplementary Table 3**
